# Possible Holt-Oram Syndrome: Missed Prenatal Diagnosis and Sub-Optimal Management in a Poor-Resourced Hospital

**DOI:** 10.4274/balkanmedj.galenos.2019.2018.12.48

**Published:** 2019-05-10

**Authors:** Ayokunle Osonuga, Jeffrey K. Arhin, Gloria C. Okoye, Adebayo Da’Costa

**Affiliations:** 1 Department of Internal Medicine, Babcock University Teaching Hospital, Ilishan, Nigeria; 2 Department of Pediatrics, Nadowli District Hospital, Upper West Region, Ghana; 3 School of Medical Sciences, Kwame Nkrumah University of Science and Technology, Kumasi, Ghana; 4 Department of Emergency Medicine, Medway NHS Foundation Trust, Kent, United Kingdom

Holt–Oram syndrome is a rare autosomal dominant condition resulting from a gene mutation on 12q24.1 (TBX5) and causes anomalies in bony segment of the upper limbs and the cardiovascular system (1). This syndrome is the most common among the six Heart–hand syndromes (2). Other differentials to be considered include Fanconi anemia, TAR syndrome, Baller Gerold syndrome, and Nager syndrome. The first case was reported in 1960; since then, more than 300 cases have been reported. The diagnosis is confirmed by TBX5 gene analysis (3,4,5).

A male baby was delivered via cesarean section at 39 weeks, on account of fetal distress (fetal heart rate <100 beats/min) and polyhydramnious, to a 40-year-old para five woman who had no history of consanguinity, antenatal morbidities, nor family history of similar presentation at birth. Cardiotocography for non-stress testing was unavailable; hence, the decision for the procedure was purely clinical.

The first obstetric scan conducted by a sonographer at 19 weeks estimated gestational age, indicated a single fetus with estimated weight of 302 g and adequate liquor (AFI=11.6 cm). Anomaly screening was not performed due to lacking expertise and facilities. A repeat obstetric scan conducted at 32 weeks of estimated gestational age showed a single fetus in breech presentation with an estimated weight of 1638g. At this time, the liquor volume increased (AFI=33.4 cm). An oral glucose tolerance test was conducted to rule out gestational diabetes as the cause of the polyhydramnious, and the result was negative. A third obstetric scan indicated complete breech presentation and an AFI of 33.7 cm (AFI percentile >95p).

Upon delivery, he was noticed to have dysmorphic features including head circumference of 32 cm with craniosynostosis. Musculoskeletal examination (Figure 1) revealed shortened right forearm and fixed flexion of both wrist joints at about 30° with inability to extend. The right forearm measured 3 cm, and the left forearm measured 6 cm in length. Both arms measured 8 cm in length. All fingers where of normal length and shape with overriding of the right index finger. The lower limbs were grossly intact.

Cardiovascular examination revealed a non-radiating grade 3 pansystolic murmur, loudest over the left sternal border. Diffuse coarse crackles were heard in all lung zones and oxygen saturation at about 65% and 94% at room air and intranasal oxygen respectively in the first 12 hours. The SpO2 level gradually declined to 34% despite an increase in oxygen delivery. Electrocardiography and echocardiography was not conducted as these were not available in the hospital. The nearest cardiothoracic center was about a two-day drive away. The index patient died the next day despite resuscitative measures.

The babygram done is shown in Figure 2. The cardiac silhouette was invisible, but the radio-opacity of the left lung zone suspected to be the heart was increased. The right forearm showed markedly shortened and curved radius and ulna (shown) compared with the contralateral side (not shown), with absent carpal bones in the right wrist. Written informed consent was obtained from the parent.

These findings emphasize the importance prenatal diagnosis, cardiovascular examination, and investigation in newborns with upper limb anomalies. The nature and extent of the cardiac defect was not concluded as relatives refused autopsy for religious reasons. The number of radiologists in rural areas is ebbing, and prenatal screening tests are expensive in sub-Saharan Africa. Clinicians need to be aware of uncommon syndromes, such as the present case, and if possible initiate referral mechanisms to centers where they can be managed.

This report highlights the diagnostic and management challenge of an uncommon Heart–hand syndrome by clinicians in a low resource setting.

## Figures and Tables

**Figure 1:**
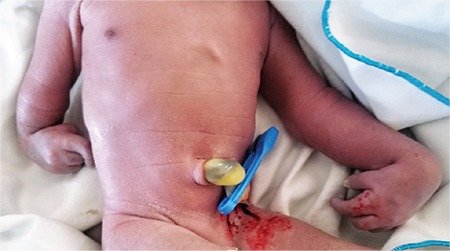
Picture showing upper limb anomaly.

**Figure 2:**
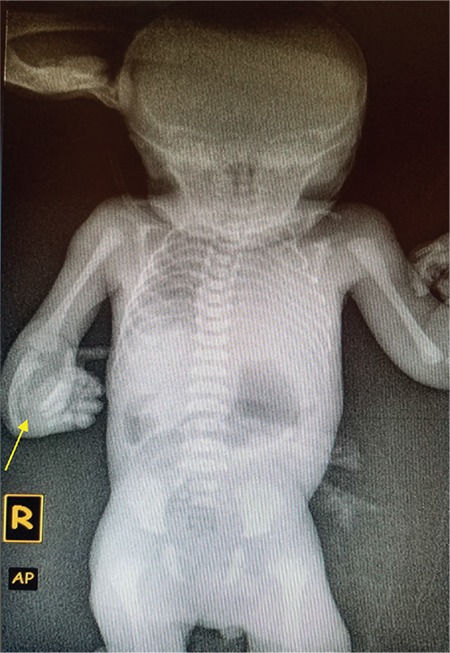
X-ray of newborn.
